# Constant Load Pedaling Exercise Combined with Electrical Muscle Stimulation Leads to an Early Increase in Sweat Lactate Levels

**DOI:** 10.3390/s22249585

**Published:** 2022-12-07

**Authors:** Tomonori Sawada, Hiroki Okawara, Daisuke Nakashima, Kaito Ikeda, Joji Nagahara, Haruki Fujitsuka, Sosuke Hoshino, Yuta Maeda, Yoshinori Katsumata, Masaya Nakamura, Takeo Nagura

**Affiliations:** 1 Department of Orthopaedic Surgery, Keio University School of Medicine, 35 Shinanomachi, Shinjuku-ku, Tokyo 160-8582, Japan; 2 Institute for Integrated Sports Medicine, Keio University School of Medicine, 35 Shinanomachi, Shinjuku-ku, Tokyo 160-8582, Japan; 3 Department of Cardiology, Keio University School of Medicine, 35 Shinanomachi, Shinjuku-ku, Tokyo 160-8582, Japan; 4 Department of Clinical Biomechanics, Keio University School of Medicine, 35 Shinanomachi, Shinjuku-ku, Tokyo 160-8582, Japan

**Keywords:** sweat lactate, blood lactate, electrical muscle stimulation, exercise

## Abstract

A novel exercise modality combined with electrical muscle stimulation (EMS) has been reported to increase cardiovascular and metabolic responses, such as blood lactate concentration. We aimed to examine the effect of constant load pedaling exercise, combined with EMS, by non-invasively and continuously measuring sweat lactate levels. A total of 22 healthy young men (20.7 ± 0.8 years) performed a constant load pedaling exercise for 20 min at 125% of the pre-measured ventilatory work threshold with (EMS condition) and without (control condition) EMS stimulation. Blood lactate concentration was measured by blood samples obtained from the earlobe every minute. Sweat lactate was monitored in real time using a sensor placed on the forearm. The sweat lactate threshold (sLT) was defined as the point of increase in sweat lactate. sLT occurred significantly earlier in the EMS condition than in the control condition. In the single regression analysis, the difference in sLT between the two conditions, as the independent variable, was a significant predictor of the difference in blood lactate concentrations at the end of the exercise (*p* < 0.05, *r* = −0.52). Sweat lactate measurement may be a noninvasive and simple alternative to blood lactate measurement to determine the effectiveness of exercise combined with EMS.

## 1. Introduction

Electrical muscle stimulation (EMS) has been widely used in rehabilitation and sports to assist in exercise therapy or to increase the exercise load, in combination with regular exercise [1,2,3,4,5,6]. Adding EMS has a significant impact on muscle metabolism and induces substantial physiological adaptations [7,8]. Stimulated contractions caused by adding EMS intensively activates anaerobic glycolysis for energy production by phosphocreatine and glycogen degradation, leading to an increase in blood lactate concentration [9,10,11]. Lactate, which is produced in the glycolytic pathway, is widely recognized as an efficient energy source used by systemic organs [12]. Additionally, it has been reported that lactate increases the peroxisome proliferator-activated receptor-γ coactivator- (PGC-) 1α mRNA expression and mitochondrial biogenesis [13,14,15,16,17]. PGC-1α is the master controller of mitochondrial biogenesis and promotes mitochondrial biogenesis through the activation of various transcription factors [18]. Since mitochondrial content is generally considered an indicator of endurance performance [19], increasing mitochondrial biogenesis induced by exercise training is believed to be an important adaptative event that improves exercise tolerance capacity [20,21]. Further, an exercise-induced increase in PGC-1α mRNA was observed above the first lactate threshold, but not below it [22]. Therefore, it is important to determine the EMS intensity that increases the blood lactate levels without fatigue, as compared to exercise without EMS, to establish an efficient program of exercise training combined with EMS. However, measuring the blood lactate levels frequently to determine the appropriate EMS output is not feasible, since it requires invasive blood sampling. Additionally, it is unclear whether the optimal measurement time should be at the moment of puncture, or when the blood is discharged after a time lag of 5–10 s following the puncture. Thus, establishing a noninvasive method of monitoring the dynamics of metabolism during exercise combined with EMS is necessary.

To date, noninvasive biosensors of lactate using sweat from the body surface have been reported [23] and have shown that sweat lactate levels increase in conjunction with exercise intensity [24,25,26]. In a recent study, the point of sweat lactate level elevation during incremental exercise was well correlated with the lactate threshold determined from the blood lactate concentration and the ventilatory threshold (VT), determined using a respiratory gas analyzer in patients with cardiovascular disease, as well as healthy individuals [27]. When this technology is applied to exercise combined with EMS, the noninvasive and continuous monitoring of lactate behavior during exercise could be possible, and increased blood lactate levels may be more easily detected by measuring sweat lactate levels. In particular, it would be suitable for the remote rehabilitation of patients and athletes outside of medical institutions and may enable effective and safe exercise based on daily physical conditions.

Thus, the current study aimed to examine whether sweat lactate concentrations could be used to detect increased blood lactate levels during exercise combined with EMS. We hypothesized that the increase in blood lactate concentration during exercise combined with EMS would be predicted, to some extent, by the increase in sweat lactate levels.

## 2. Materials and Methods

### 2.1. Participants

After recruiting participants from one university starting in May 2021, 22 healthy recreationally trained men (average age, 20.3 years) participated in this study conducted between June and September 2021 (Table 1). The inclusion criteria were (1) age ≥18 years and (2) no medical history of illness or injury, and not currently taking any medication. The exclusion criteria were (1) lower extremity injury or disorder that hinders complete participation in exercise, (2) metabolic, cardiac, respiratory, and psychiatric diseases, and (3) severe skin disease. The study protocol was conducted in compliance with the ethical guidelines for medical and health research involving human subjects and was approved by the Institutional Review Board of our institution (approval number: 20190229). Written informed consent was obtained from the individuals for study participation and publication of the findings before enrollment.

### 2.2. Experimental Protocol

All participants were asked to visit our laboratory three times. For each of the three visits, an interval of at least 3–14 days was allowed. In all three sessions, the room temperature was set to the same level and pedaling exercises were performed using an electromagnetically braked ergometer (POWER MAX V3 Pro; Konami Sports Co., Ltd., Tokyo, Japan) with a target of 70 rpm. At the first visit, prior to an incremental load test, body composition was measured using a multi-frequency body composition meter (MC780A-N; TANITA Corporation, Tokyo, Japan). Then, the exercise test was performed, and the VT was determined for each individual with a respiratory gas analyzer using the ventilatory equivalent, excess carbon dioxide, and modified V-slope methods [28]. Specifically, following a 2 min rest to stabilize the heart rate and respiration, the participants performed a 4 min warm-up, pedaling at 20 W, and then exercised at increasing intensity until they could no longer maintain the pedaling rate (volitional exhaustion). The resistance was increased in 25 W increments from 50 W at 1 min intervals. Once the exercise tests were terminated, the participants were instructed to stop pedaling and remain on the ergometer for 3 min. The expired gas flow was measured using a breath-by-breath automated system (Aeromonitor^®^; Minato Medical Science Co., Ltd., Osaka, Japan). Prior to the second and third visits, the participants were instructed to keep a fast for 3 h prior to the measurements and to refrain from caffeine and alcohol intake and engaging in intensive exercises within 12 h. Participants were also asked to drink 500 mL of water before the exercise. On the second visit, constant load pedaling exercise, without EMS stimulation (control condition, hereinafter called “CR condition”), was performed. To perform the exercise, the sweat lactate level was monitored with a sweat lactate sensor (Grace Imaging Inc., Tokyo, Japan) attached to the left forearm, and a Fitbit Inspire HR (Fitbit Inc., San Francisco, CA, USA) was attached to the left wrist, two-finger widths above the ulnar styloid process, to measure the heart rate. After an initial 2 min of rest and measurement of sweat and blood lactate levels, a 20 min pedaling exercise was performed at a constant load of 125% of the pre-measured ventilatory work threshold. The loading of VT125% was determined based on a previous study [11] reporting that the difference in blood lactate concentrations between conditions with and without EMS was significantly greater in the VT125% exercise loading than in the VT50% and VT75% exercise loading. During the pedaling exercise, the sweat lactate level was measured at 1 Hz, and the blood lactate level was measured (Lactate Pro 2; ARKRAY, Inc., Kyoto, Japan) by drawing blood from the earlobe every minute. The exercise was stopped under the following conditions, even if the duration was <20 min: when the heart rate exceeded 190 bpm; when the examiner judged the exercise to be dangerous to the patient; when the participant requested to stop; when the participant became exhausted; or when it became difficult to maintain the target speed of 70 rpm. In such cases, blood lactate levels were measured at the end of the exercise. At the third visit, the exercise was performed in the same way as the second visit, but with EMS stimulation. In the EMS condition, participants wore a commercially available EMS suit separated into a top and a bottom section (Powersuit; MTG Ltd., Nagoya, Japan) and performed pedaling exercises while seven muscles on each side of the upper and lower trunk were stimulated (biceps brachii, triceps brachii, rectus abdominis, oblique abdominis, gluteus medius, quadriceps femoris, and hamstrings). In accordance with a previous study [11], all electrode pairs were synchronized and biphasic square current pulses with a 100 μs duration were constantly applied at a stimulation frequency of 4 Hz. The EMS intensity was set to a maximum intensity at which each participant did not feel pain or discomfort. The maximal electrical potential and current of this device were 50 V and 4.85 mA, respectively.

### 2.3. Sweat Lactate Threshold Measurements

The sweat lactate level was measured using a wearable sensor, which quantifies lactate concentration as a current value because it reacts with sweat lactate and generates an electric current [27]. The current value can be obtained as continuous data within 0.1–80 μA in 0.1-μA increments. After calibration using saline for approximately 3 min, the sensor chip connected to the sensor device was attached to the participants’ dorsal left forearm, which was cleaned with an alcohol-free cloth. Additionally, the data were recorded at a sampling frequency of 1 Hz for mobile applications using a Bluetooth connection. The recorded data were converted to moving average values over 13 s intervals and individually underwent zero correction using the baseline value. Sweat lactate threshold (sLT) was defined as the first significant increase in the sweat lactate level above baseline, based on graphical plots [27] by three researchers in consultation.

### 2.4. Statistical Analysis

All data are presented as the mean plus standard deviation. For the heart rate and blood lactate concentration data, in a population who performed 20 min of exercise in both conditions, two-way analysis of variance with repeated measures was used to test the main effects under two conditions (CR and EMS) and five time points (baseline, 5, 10, 15, and 20 min), as well as the interaction effect between the time point and the condition. Bonferroni correction was performed for post hoc pairwise comparison. For sLTs, paired t-tests were conducted on participants for whom sLTs could be defined in order to compare values between the two conditions. Additionally, to predict the increase in blood lactate concentration in the EMS condition relative to the CR condition, a single regression analysis was performed, with the blood lactate concentration change between the two conditions as the dependent variable, and the change in the time to reach sLT between the two conditions as the independent variable. In the regression analysis, the blood lactate concentration was used as the value after 20 min, or at the end of exercise, because the analysis included patients who could not exercise for 20 min. All statistical analyses were performed using SPSS Statistics version 27.0 (IBM Corp., Armonk, NY, USA), with statistical significance set at 0.05.

## 3. Results

**Figure 1:**
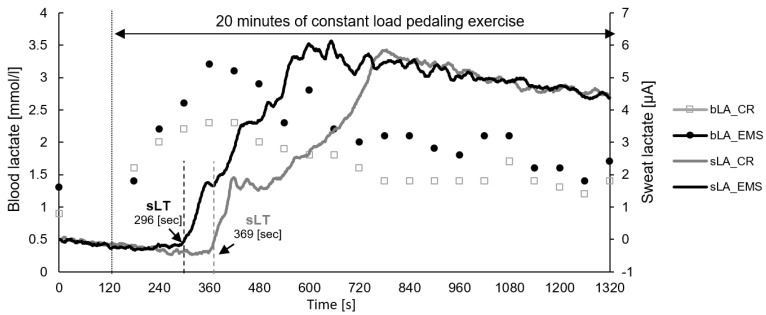
Representative data of sweat lactate and blood lactate concentrations during constant load pedaling exercise. EMS, electrical muscle stimulation; CR, without EMS stimulation; bLA, blood lactate; sLA, sweat lactate; sLT, sweat lactate threshold.

Representative data of sweat and blood lactate concentrations during a constant load pedaling exercise are shown in Figure 1. Of the 22 participants, 15 completed 20 min of constant load exercise in both conditions, and the remaining 7 had difficulty maintaining the target speed of 70 rpm due to fatigue and stopped the exercise before 20 min. Four of the seven participants had difficulty completing 20 min in both conditions, two had difficulty completing 20 min only in the EMS condition, and one had difficulty completing 20 min only in the CR condition. Additionally, detecting sLT was difficult in 2 out of 22 participants, as the sensor did not respond, due to lack of sweating in one participant, and the sensor chip was poorly connected in the other participant. Therefore, only 20 participants were included in the analysis of sLT.

**Figure 2:**
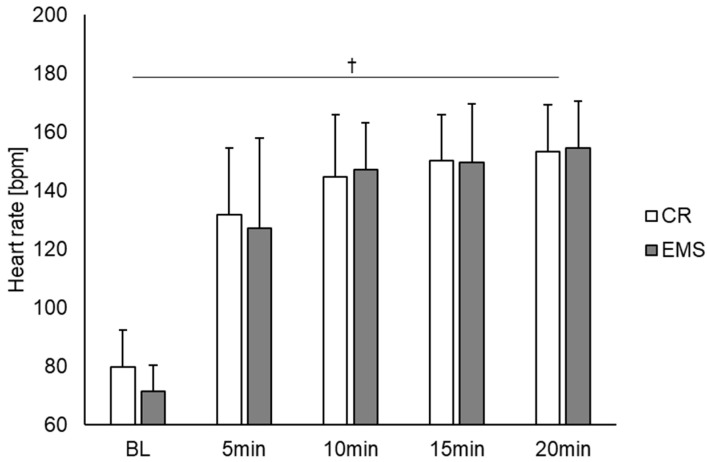
Comparison of the heart rate between the EMS and CR conditions (*n* = 15). †: *p* < 0.01 significant main effect at time point. EMS, electrical muscle stimulation; CR, without EMS stimulation.

**Figure 3:**
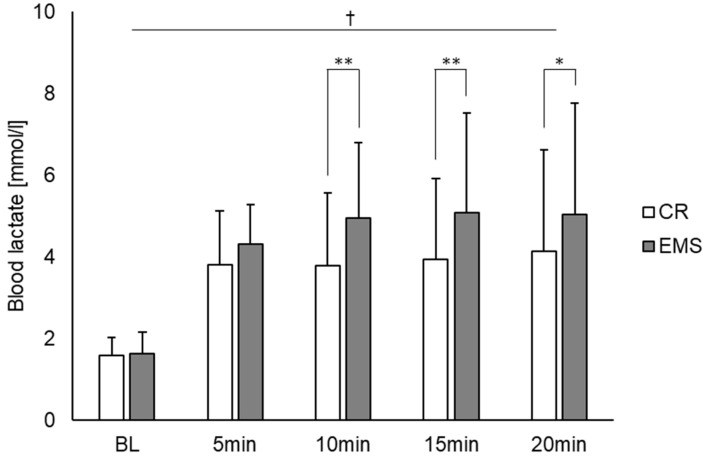
Comparison of blood lactate concentrations between the EMS and CR conditions (*n* = 15). †: *p* < 0.01 significant main effect at time point. * *p* < 0.05, ** *p* < 0.01 significant difference between conditions. EMS, electrical muscle stimulation; CR, without EMS stimulation.

Figure 2 shows the comparison of the heart rate under the two conditions. As a result, there was a main effect of time point observed [F (4, 56) = 167.831, *p* < 0.01]. Post hoc test results showed significant differences between BL and other measurement time points (5, 10, 15, and 20 min), between 5 and 10, 5 and 15, and 5 and 20 min. Contrastingly, there was no main effect for condition, and no interaction effect between condition and measurement point. Figure 3 also depicts the comparison of blood lactate concentration between the two conditions. For blood lactate concentration, there was a main effect of time point [F (4, 56) = 19.703, *p* < 0.01] and condition [F (1, 14) = 11.050, *p* < 0.01] and an interaction effect between time point and condition [F (4, 56) = 4.069, *p* < 0.01]. Post hoc test results showed significant differences between BL and other measurement points (5, 10, 15, and 20 min). Additionally, significant differences were observed between the conditions at 10, 15, and 20 min, with the EMS condition showing an increase in blood lactate concentration compared to the CR condition.

**Figure 4:**
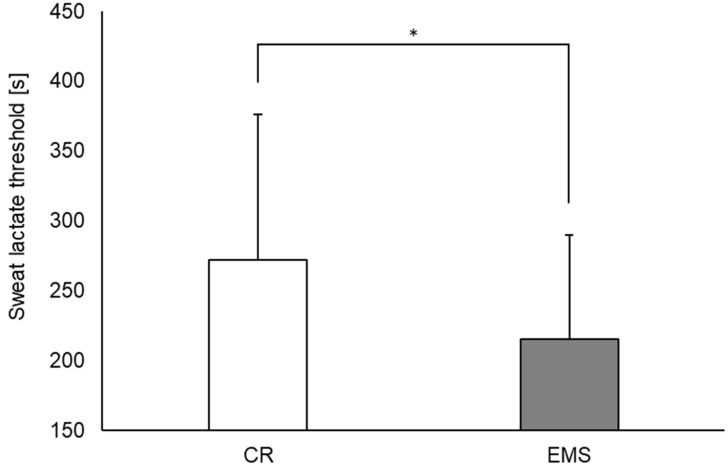
Comparison of sweat lactate threshold between the EMS and CR conditions (*n* = 20). * *p* < 0.05. EMS, electrical muscle stimulation; CR, without EMS stimulation.

**Figure 5:**
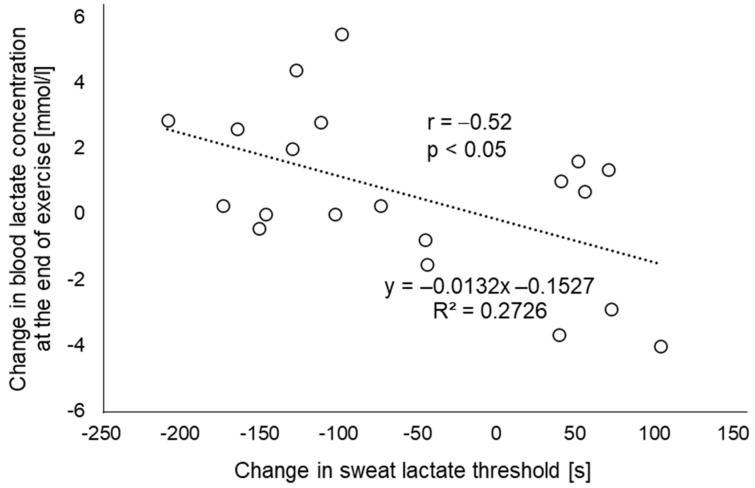
Relationship between the change in blood lactate concentration at the end of exercise and the change in sweat lactate threshold (*n* = 20). The amount of change is calculated as the value of the EMS condition minus the value of the CR condition. EMS, electrical muscle stimulation; CR, without EMS stimulation.

Regarding sLT, it occurred significantly earlier in the EMS condition than in the CR condition (Figure 4, 215.2 ± 74.5 s and 271.8 ± 104.3 s, respectively; *p* < 0.05). Additionally, in the single regression analysis, the difference in sLT between the two conditions as an independent variable was a significant predictor of the difference in blood lactate concentrations at the end of the exercise (Figure 5, *p* < 0.05; *r* = −0.52).

## 4. Discussion

The primary result of this study was that the increase in blood lactate concentration due to exercise combined with EMS could be explained, to some extent, by the sLT changes, which can be measured noninvasively. Previous studies have reported that exercise combined with EMS leads to an increase in blood lactate concentration [9,10,11]. The mechanism of increased blood lactate concentration is considered to be due to the recruitment of high-threshold motor units and muscle fibers by the additional use of EMS [29,30,31,32]. Therefore, to accurately assess the load imposed on the skeletal muscle by exercise combined with EMS, it was necessary to frequently measure lactate concentration, which is considered to directly reflect an increase in metabolic rate and glycolytic carbon flow in the skeletal muscles [33]. Additionally, as lactate is also considered a signal molecule that induces mitochondrial neogenesis in skeletal muscle cells [14], it is beneficial to determine the EMS loads that increases blood lactate concentration in each individual. However, measuring the blood lactate concentration was not feasible because it required blood sampling. Thus, we focused on the lactate contained in sweat. Sweat lactate measurement has the potential to compensate for the disadvantages of conventional evaluation methods, as it can be measured noninvasively and easily, and this could be demonstrated through exercise combined with EMS. The sweat lactate device that we used is capable of measuring sweat lactate continuously and over a long period of time by adjusting the thickness and composition of the topcoat applied to the upper layer of lactate oxidase on the sensor chip [27]. Therefore, it is able to monitor lactate level changes without deactivation of the enzyme in a single measurement. Our results suggest that this device may be applied in the future for setting EMS loads and/or determining the effectiveness of exercise in various training environments, such as gyms, outdoors, and at home.

As for sweat lactate, it reportedly does not reflect the blood lactate levels during exercise [34,35]. While lactate is produced in sweat reflecting exercise intensity, it is influenced by the body’s production of lactate, the rate of sweating, and metabolic kinetics in the sweat glands [34,36]. On the other hand, treating sweat lactate as an elevated point during incremental load exercise has been verified in a previous report to be consistent with LT obtained from blood [27]. One possible reason for this measurement consistency is that increased lactate production from the muscle cells reflecting LT may induce a simultaneous increase in sweat lactate values through changes in autonomic balance, hormones, acid-base equilibrium, and metabolic dynamics [37,38]. Although the exercise protocol used in the current study was a constant load rather than an incremental load, because of the load above AT (VT125%), the lactate discharged from the sweat glands is thought to strongly reflect anaerobic metabolism by the skeletal muscle, in addition to sweat gland metabolism. In such a load setting, blood and sweat lactate concentrations increase in the CR condition itself, which would likely increase further when combined with EMS. In our results for the single regression analysis, R^2^ was 0.2726, and in terms of correlation coefficient, r = −0.52. Therefore, our results showed a moderate association between the increase in blood lactate at 20 min (or the end of exercise) due to the addition of EMS and the time change to the point of sLT, and the amount of change in the blood lactate concentration between conditions could be explained by the amount of change in the point of sLT. More recently, there have been reports on the development of lactate biosensors that are not affected by sweat secretion rate [39] and on the development of systems that combine colorimetric analysis with deep learning to detect sweat lactate [40]. These techniques may contribute to more accurate sweat lactate measurement during exercise. Moreover, the results of this study suggest that noninvasive skeletal muscle metabolic assessment, which previously required blood sampling, may be possible with sweat lactate.

Our results showed that there was no difference in the heart rate between the two conditions. A previous study reported a difference between the conditions with and without EMS at 80% VT loading [9], which was inconsistent with the results of this study. One possible reason for the inconsistency in the results could be the difference in the exercise load intensity. Watanabe et al. examined the effects of different voluntary exercise intensities (50%, 75%, 100%, and 125% of VT) on metabolic responses to exercise combined with EMS and reported that the increment in oxygen consumption at 125% of VT was significantly lower than those at lower exercise intensities [11], suggesting that the additional recruitments of motor units associated with EMS would be attenuated during high-intensity voluntary exercise, especially over the AT. Contrarily, EMS can induce a greater reliance on anaerobic glycolysis for energy production, along with phosphocreatine degradation and lactate formation [41,42,43,44]. In this study, the blood lactate concentration was also increased during exercise combined with EMS. Therefore, the constant load exercise combined with EMS at 125% of VT may have further increased the glycolytic metabolism, through large and fatigable fast-twitch motor units with glycolytic fibers. Although future investigations should be conducted to include exercise intensities of 80% VT and 100% VT, as in previous studies, there may be a limited increase in heart rate with the addition of EMS under exercise intensities above AT, in which lactic can accumulate.

This study has several limitations. First, all 22 study participants were university students and male, suggesting a population bias. Therefore, future evaluations including participants with different characteristics, such as age, sex, and exercise capacity, would allow for a wider interpretation and application of the results. Second, it was difficult to measure the heart rate using an electrocardiogram at the chest due to wearing a pair of upper and lower full-body suits. Therefore, measurements were taken from the peripheral wrist with a Fitbit instead. Thus, it may be necessary to consider the influence of measurement uncertainty [45,46] as a reason why there was no difference between the two conditions with respect to the heart rate. Third, the EMS load intensity was set based on the subjectivity of everyone; thus, the stimulus intensity differed among participants. Fourth, we cannot eliminate the possibility that the participants were not given any control, such as fasting or prohibiting exercise on the previous day, which may have affected the pattern of blood lactate level changes. Therefore, it is considered that such controls are also necessary to conduct more precise experiments.

## 5. Conclusions

Constant load pedaling exercise combined with EMS resulted in an early increase in sweat lactate levels, which could explain, to some extent, the increase in blood lactate concentrations. Since sweat lactate measurement is noninvasive, continuous, and easy to perform, it may be expected to be used as an alternative to blood lactate measurement for monitoring metabolism during exercise combined with EMS and for determining its effectiveness in the future.

## Figures and Tables

**Table 1:** Participant characteristics (*n* = 22).

	Mean (SD)	Range
Age (years)	20.7 (0.8)	19–22
Height (cm)	174.0 (5.4)	161.0–184.0
Weight (kg)	66.8 (9.0)	46.8–91.5
BMI (kg/m^2^)	22.0 (2.2)	18.1–27.0
Body fat ratio (%)	15.4 (4.3)	8.6–23.8
Fat mass (kg)	10.5 (4.0)	4.0–20.1
Lean body mass (kg)	56.4 (6.2)	42.8–71.4
Muscle mass (kg)	53.4 (5.9)	40.5–67.7
Total body water (kg)	40.5 (6.5)	30.3–57.6
Body water (%)	59.7 (4.7)	49.6–65.9

BMI, body mass index.

## Data Availability

The data that support the findings of this study are available from the corresponding author upon reasonable request.
